# Stratification of co-evolving genomic groups using ranked phylogenetic profiles

**DOI:** 10.1186/1471-2105-10-355

**Published:** 2009-10-27

**Authors:** Shiri Freilich, Leon Goldovsky, Assaf Gottlieb, Eric Blanc, Sophia Tsoka, Christos A Ouzounis

**Affiliations:** 1 The Blavatnik School of Computer Sciences and School of Medicine, Tel-Aviv University, Tel-Aviv 69978, Israel; 2 Computational Genomics Unit, Institute of Agrobiotechnology, Centre for Research & Technology Hellas, (CERTH), GR-57001 Thessalonica, Greece; 3 School of Physics and Astronomy, Tel-Aviv University, Tel-Aviv 69978, Israel; 4 King's College Centre for Bioinformatics (KCBI), School of Physical Sciences & Engineering, King's College London, Strand, London WC2R 2LS, UK; 5 MRC Centre for Developmental Neurobiology, New Hunt's House, King's College London, Guy's Campus

## Abstract

**Background:**

Previous methods of detecting the taxonomic origins of arbitrary sequence collections, with a significant impact to genome analysis and in particular metagenomics, have primarily focused on compositional features of genomes. The evolutionary patterns of phylogenetic distribution of genes or proteins, represented by phylogenetic profiles, provide an alternative approach for the detection of taxonomic origins, but typically suffer from low accuracy. Herein, we present *rank-BLAST*, a novel approach for the assignment of protein sequences into genomic groups of the same taxonomic origin, based on the ranking order of phylogenetic profiles of target genes or proteins across the reference database.

**Results:**

The rank-BLAST approach is validated by computing the phylogenetic profiles of all sequences for five distinct microbial species of varying degrees of phylogenetic proximity, against a reference database of 243 fully sequenced genomes. The approach - a combination of sequence searches, statistical estimation and clustering - analyses the degree of sequence divergence between sets of protein sequences and allows the classification of protein sequences according to the species of origin with high accuracy, allowing taxonomic classification of 64% of the proteins studied. In most cases, a main cluster is detected, representing the corresponding species. Secondary, functionally distinct and species-specific clusters exhibit different patterns of phylogenetic distribution, thus flagging gene groups of interest. Detailed analyses of such cases are provided as examples.

**Conclusion:**

Our results indicate that the rank-BLAST approach can capture the taxonomic origins of sequence collections in an accurate and efficient manner. The approach can be useful both for the analysis of genome evolution and the detection of species groups in metagenomics samples.

## Background

The notion of genes as components of a genome has been recently challenged with the advent of metagenomics, where the phylogenetic origin of entire gene pools is not necessarily known [1]. Particular environments and biological symbioses establish crucial constraints on the nature of genes that can be associated with the species communities under investigation [2]. Such constraints can be used to infer the number and types of species that contribute to these gene pools. In computational terms, the goal is to detect unambiguous and unique intra-genomic signals that can be used as signatures for the association of gene collections assigned to species groups.

Several approaches have been proposed to detect genomic signatures on the basis of nucleotide composition [3-5]. These approaches enable, to a varying degree of accuracy, the species classification of genes according to their compositional signatures, and their association with phylogenetic or environmental factors [6-8]. More recently, these methods have been applied to environmental sequencing samples, in order to detect the origins of these sequence fragments [9,10].

Whereas the aforementioned methods focus on the detection of structural constraints of genes, another family of methods appears to be ideally suitable for this endeavor, using the detection of gene distribution patterns across taxa [11]. These evolutionary signatures can be captured by phylogenetic profiles, the binary representation of the taxonomic distribution of genes (presence or absence) across genome [12]. Phylogenetic profiles thus represent the evolutionary history of genes and genomes, constrained by functional properties in particular environments, and can be used to understand both the structural and evolutionary properties of a genome at the gene level. In the case of metagenomics, desirable properties of such an approach include the detection of genes with common co-inheritance patterns, histories, and thus origins. Given that the major factors shaping gene content are gene loss, gene genesis and horizontal gene transfer, [13] it would also be desirable to trace unique intra-genomic signals with respect to those factors.

**Figure 1:**
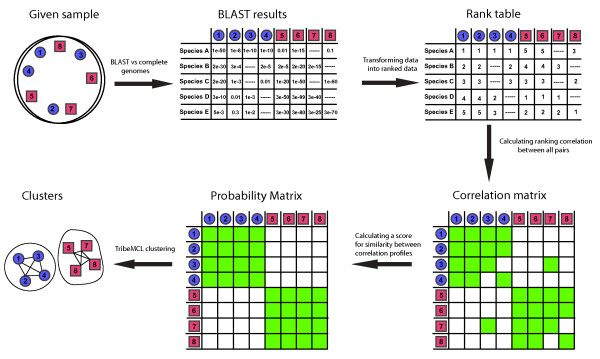
**The rank-BLAST classification procedure**. The colored circles and squares represent proteins; different shapes and colors represent different taxonomic origins. Protein sequences lacking taxonomic-annotations (retrieved for example form metagenomic samples which include partial or complete genome sequences of assortments of species) are subject to a BLAST search. For each protein, the results of the BLAST search are converted into a vector describing the ranking order of species where it recognizes homologues. Each species is ranked once, according to its first appearance. All possible protein-pairs combinations are compared in order to determine whether the positions of species on the vectors are correlated. Two vectors are considered to be correlated (green squares) when their Kendall tau correlation coefficient is higher than a threshold (see **Methods**). The correlation matrix is transformed into a probability matrix, estimating the significance of the similarity between the correlation profiles of each protein pair. Green boxes represent protein pairs where the P value is lower than a threshold (see **Methods**). In the final stage, proteins are clustered according to the similarity of their probability vectors.

Here, we describe the rank-BLAST classification approach for tracing an intra-genomic signal. The rank-BLAST classification is an elaborate interpretation and data manipulation procedure to capture sequence similarity relationships from BLAST searches [14]. BLAST searchs report the degree of sequence divergence between query and reference proteins. In cases where the taxonomic origin of a query protein is not known, the extent of its divergence from the reference proteins (for which taxonomic origins are known) can serve to delineate the genomic classification of the input proteins. The general concept behind the approach (illustrated in Figure 1) is to use the ranking order of species in which BLAST detects homologues, in order to construct a profile common and unique to taxonomically-related genes. Several factors make the rank-BLAST approach appropriate for delineating intra-genomic signal while reducing the noise caused by the differences in the histories of genes within a genome. First, rather than using the actual level of similarity, comparing the relative order of species in a vector reduces the effect of the differences in substitution rates between proteins, where some proteins are known to evolve at a faster rate than others (as demonstrated by proteins 1 and 2 in Figure 1). Second, limiting the comparison to the set of species common to the two vectors compared eliminates discrepancies derived from a species-specific gene loss in a given sample (as demonstrated by proteins 3 and 4 in Figure 1). Whereas gene loss and differences in the rate of evolution are not expected to eliminate the intra-genomic signal, recent events of gene genesis and lateral gene transfer are expected to degrade the similarity signal of the corresponding genes. Such genes will carry an anomalous inheritance signal and are of special interest since they may represent recent adaptations in the genome of a species [15].

In this work, we present the rank-BLAST classification procedure, estimate the strength and accuracy of the classification using a synthetic dataset, and discuss its biological relevance. First, we describe the choice of the optimal parameters for detecting an intra-genomic signal while allowing inter-genomic separation. Second, we describe the clusters formed and classify them with respect to taxonomically-expected inheritance signal or an anomalous inheritance signal. Third, we identify and characterize a group of proteins carrying an anomalous inheritance signal, in order to better understand the biological significance of the ranked phylogenetic profiles.

## Results

### Clustering proteins into genomic-groups according to their rank-BLAST profile

The first stage in tracing the intra-genomic signal is to assess how similar are the rank-BLAST profiles of proteins with varying degrees of taxonomical relatedness. Two main considerations guided the choice of the species tested. First, species have to represent several phylogenetic proximities. Second, comparing a large number of protein-pair combinations is an exhaustive computational process and it is thus effective towards the study of species with a small number of proteins. The low sequence and function redundancy which is characteristically observed in small-size proteomes [16-20] is an additional advantage of these species in terms of improving the efficiency of the practice. The use of larger genomes, comprising many duplicated proteins, is expected to yield redundant profiles, unnecessarily increasing the complexity of the computational process.

In accordance with the above considerations, we have chosen to perform this analysis for the proteome of *Mycoplasma genitalium *- the smallest cellular species that has been sequenced [21] - and four additional species with small genomes at varying degrees of phylogenetic relatedness to *M. genitalium*: an organism from the same family; one from the same phylum; another one from the same super-kingdom; and one from a different super-kingdom. From each category (phylogenetic distance) we selected the fully sequenced species with the smallest number of proteins. The full list of species is given in Table 1.

**Table 1:** Taxonomic classification and number of proteins of the fully sequenced species analyzed.

**Species**	**Taxonomy†**	**Taxonomic relatedness to *M. genitalium***	**Evaluation of the divergence distance from *M. genitalium*‡**	**Number of proteins**
*Mycoplasma genitalium*	**Bacteria; Firmicutes; Mollicutes; Mycoplasmatales; Mycoplasmataceae; Mycoplasma**	-	0	480
*Ureaplasma parvum*	**Bacteria; Firmicutes; Mollicutes; Mycoplasmatales; Mycoplasmataceae; **Ureaplasma	Within family	17	611
*Streptococcus pyogenes*	**Bacteria; Firmicutes; Bacilli; **Lactobacillales; Streptococcaceae; Streptococcus	Within phylum	25	1696
*Buchnera aphidicola*	**Bacteria; **Proteobacteria; Gammaproteobacteria; Enterobacteriales; Enterobacteriaceae; Buchnera	Within superkingdom	31	574
*Nanoarchaeum equitans*	Archaea; Nanoarchaeota; Nanoarchaeum	From distinct super kingdoms	59	536

† The taxonomic classification is according to . The common path between each and *M. genitalium *is shown in bold.

‡ The evaluated divergence distance aims to provide a quantitative assessment of the taxonomic distance. The values are derived from the scores of the pair-wise alignments between the 16S RNA from each species and the 16S RNA from *M. genitalium *(see **Methods**). The same divergence order was obtained when distance matrix was retrieved from greengenes - a 16s RNA gene database [38] (divergence distance scores ordered as in the table: 0, 0.14, 0.25, 0.31, 0.63, see **Methods**).

**Figure 2:**
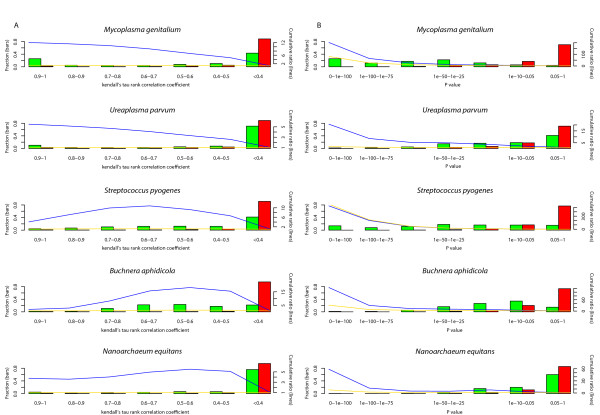
**Distributions of intra- and inter-genomic similarity scores and their ratios**. Intra-genomic combinations are all combinations between proteins common to a single genome (green bars); inter-genomic combinations are all the combination between proteins from one species to proteins from the four other species (red bars). The y-axis on the right of each plot shows the fraction of combinations which fall within a given range, where all green bars sum to 1 and red bars sum to 1. The blue and gold lines show the ratio between the cumulative fractions of inter- to inter-genomic combination. The y-axis on the left of each plot corresponds to the blue line; the gold line corresponds to a unified scale in all graphs (0 to 500). (A) The distributions of the tau rank correlation coefficients calculated between the rank-BLAST profiles of pair combinations. (B) The distributions of the P values for the hypergeometric probability calculated for the correlation-profiles of pair combinations.

For each protein coming from the set of selected species, we have constructed its rank-BLAST profile (see **Methods**) - i.e., a vector containing the order of species in which the protein recognizes homologues. For all possible protein-pair combinations, we estimated the similarity of their rank-BLAST profile by calculating the Kendall tau rank correlation coefficient - a measure for the degree of correspondence between two rankings (see **Methods**). The distributions of the correlations found in intra- and inter-genomic pair combinations are compared in Figure 2A. As expected, intra-genomic combinations result in a higher mean similarity score than the inter-genomic correlation. However, the intra- and inter-genomic distributions have a significant overlap in their distribution ranges, where the highest relative enrichment of intra-genomic combinations (i.e., the highest ratio between the fraction of intra-genomic pairs) and the fraction of inter-genomic pairs is less than one order of magnitude (observed for tau > 0.7, Figure 2). Therefore, the correlation between the rank-BLAST profiles of two proteins is by itself insufficient in order to predict whether those proteins are encoded by the same genome.

One way of maximizing the information obtained from the rank-BLAST profile is, instead of using the direct correlation measured between two proteins, to compare the pairwise similarities in the correlation profiles of proteins. The rationale behind this approach is that in cases where a small number of common observations (e.g., proteins 3 and 7 in Figure 1) results in a high correlation which does not necessarily reflect the full set of observations of each protein in the pair, the differences in their correlation profile will mask out the inter-genomic signal. In cases where we miss an intra-genomic signal due to lack, or a small number, of common observations (e.g., proteins 3 and 4 in Figure 1), the similarity in their correlation profile will reveal the intra-genomic signal. To evaluate whether two correlation profiles exhibit a significant similarity, we calculated the hypergeometric probability [22] (see **Methods**). The correlation matrix is thus transformed into a probability matrix (Figure 1).

The distributions of the hypergeometric probability values found in intra- and inter-genomic pair combinations are compared in Figure 2B. For *P *values smaller than 1 × 10^-75^, the ratio between the fraction of intra-genomic pairs and inter-genomic pairs ranges between 9 (in *Ureaplasma parvum*) to 200 (in *Streptococcus pyogenes*) - much higher than the ratio observed between the intra- and inter-values of the pure correlation (Figure 2B).

**Figure 3:**
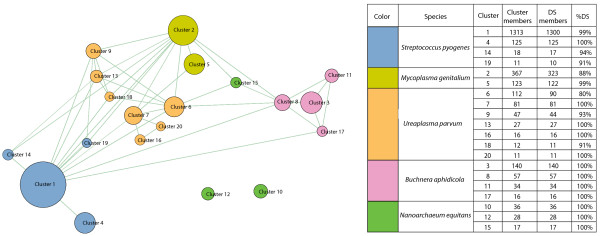
**The size and species-specificity of the 20 largest genomic groups**. Each genomic group is represented by a circle. The color of each circle corresponds to the genomic origin of most cluster members and the size of each circle corresponds to the number of cluster members, as listed at the adjacent table (right). The taxonomic-specificities of the genomic groups are indicated at the table, providing the number and fraction of proteins in a cluster which belong to the corresponding Dominant Species (DS). The clusters were constructed using the MCL algorithm (**Methods**). Layout and network construction were performed using the Biolayout software [42].

The distribution of the hypergeometric probability values therefore better stratifies the intra-genomic and inter-genomic calls and allows rediscovering the intra-genomic relations. A network was formed by linking all protein-pairs which scored a *P *value lower than 1 × 10^-75^(Figure 1), nodes in the network represent proteins and edges represent all links lower than the set threshold. This network is composed of 2748 protein-components which constitute 71% of proteins from the selected 5 species in the synthetic dataset (Table 2). Using the MCL algorithm, the protein members of the network were clustered into 63 groups of varying size, ranging from 2 to 1313 components (see **Methods**). Since almost all clusters (95%) are highly dominated by a single species (the dominant species corresponds to at least 80% of the cluster components), these are referred to as 'Genomic Groups'. Attempts to reduce the number of groups, either by reducing the granularity or by using alternative clustering approaches, resulted in non species-specific clusters [see Additional file 1]. The 20 largest clusters, with more than 10 protein-components, are illustrated in Figure 3. In all these genomic groups, one dominant species corresponds to at least 80% of the cluster components, and more than a half of the clusters are entirely species-specific.

**Table 2:** The efficiency of the rank-BLAST clustering procedure for the re-construction of genomic groups.

**Species**	**No. proteins classified into a cluster‡**	**No. proteins classified into a corresponding genomic group†**	**% of the proteins in a species (out of the complete proteome) classified into a genomic group**	**No. of genomic groups dominated by the species†**
Total	2748 (71%)	2505 (91%)	64%	20
*M. genitalium*	454 (95%)	445 (98%)	92%	2
*U. parvum*	367 (60%)	280 (76%)	46%	7
*S. pyogenes*	1522 (90%)	1452 (95%)	86%	4
*B. aphidicola*	302 (53%)	247 (82%)	43%	4
*N. equitans*	102 (19%)	81 (79%)	15%	3

‡ In brackets: the percentage out the total number of proteins in the species (or in the 5 species in the first row), as shown in **Table 1**.

† Genomic groups are clusters with at least 10 members. Their size and content is described in Figure 3. In brackets: the percentage out the number of proteins classified into a cluster.

The efficiency of the clustering procedure in re-constructing genomic groups varies between the different species (Table 2). The highest fraction of proteins classified into a large cluster is observed in *Mycoplasma genitalium*, where 92% of its protein members are classified into two genomic groups. The lowest fraction is observed in *Nanoarchaeum equitans*, where only 15% of the proteins are classified into 3 genomic clusters. It is evident that the underlying dataset of both target and database proteins plays a significant role in providing the necessary contrast for the detection of species-specific genomic groups. Using different parameters for ranking, cut-offs for the *P *value of the hypergeometric distribution, and inflation values for the clustering procedure has resulted in a similar pattern of clusters, as discussed above (not shown).

Overall, using the rank-BLAST procedure (as illustrated in Figure 1) we have succeeded in correctly clustering 64% of the proteins in the analysis into consistent, species-specific genomic groups (Table 2). In comparison, only 2180 out of the 3891 proteins (56%) had recognized a BLAST best-hit partner within the same genus indicating that the rank-BLAST procedure is more sensitive than a simple BLAST search (Methods). The protein NEQ108 (a tRNA methyltransferase) from the archaeal species *Nanoarchaeum equitans *provides an example for a protein with a distant best-hit partner (a tRNA methyltransferase from the bacterial species *Aquifex aeolicus*) which is classified by the rank-BLAST procedure into a corresponding genomic group (cluster 12 - a cluster clearly dominated by *Nanoarchaeum equitans' *proteins Figure 3): although its best-hit partner is a bacterial protein, the following hits of NEQ108 are proteins from the archaeal superkingdom and hence the rank-BLAST approach, taking into account all hits rather than only the best-hit partner, classifies this protein together with other *Nanoarchaeum equitans' *proteins. A few examples exist for proteins with a best-hit partner from a closely-related species which are classified into a non-corresponding genomic group. The *Mycoplasma genitalium *protein MG014 (Putative ABC transporter ATP-binding protein) for example recognizes a best-hit partner from *Mycoplasma pneumoniae *- another member of the Mycoplasma genus - yet it is classified into a genomic group dominated by *Streptococcus pyogenes *proteins (cluster 1). Notably, although its best-hit partner is another *Mycoplasma *protein, its following hits are to proteins from Clostridia species (including *Caldicellulosiruptor saccharolyticus*, *Anaerocellum thermophilum*, *Clostridium tetani*). However, overall, the genomic groups are highly specific with 97% of the proteins classified into corresponding genomic groups (Methods). The correspondence between the order of species in the rank-BLAST profile and their taxonomic proximity to the relevant species is further discussed in the following paragraphs.

### The main genomic groups carry a signal which reflects the vertical evolution of the corresponding species

The primary goal of the rank-BLAST classification procedure is to capture a signal reflecting the inheritance pattern of the target genes. Therefore, genomic clusters were characterized in order to examine whether such a signal is carried by the clusters as a whole. Since we retrieved more than a single genomic cluster for each species, we first aimed to characterize the *main *genomic group of each species, i.e., a cluster where the co-inheritance pattern of its member proteins matches the known phylogeny of the organism.

**Figure 4:**
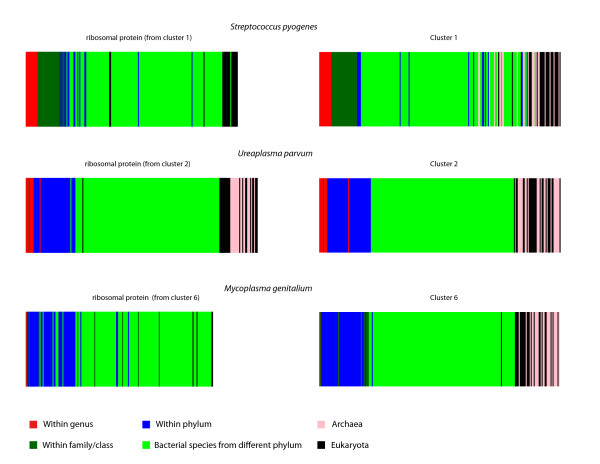
**Barcode representations of the rank-BLAST vector of three conserved ribosomal proteins and their corresponding clusters**. The rank-BLAST profile of each cluster was constructed by calculating the mean position in the cluster of each of the 243 database species (i.e., for each database species we calculated its mean position in the individual vectors of all protein-members of the cluster). Each line in the barcode represents a species. The color code represents the phylogenetic proximity between the species in the vector (database species) and the species dominating the cluster (listed on top), i.e., the species coding the majority of the proteins in the cluster (specificity of the clusters is described at Figure 3). The 50S ribosomal protein L27 (rpl27) is the conserved protein from cluster 1. The 50S ribosomal protein L2 (rpl2) is the conserved protein from cluster 2. The 50S ribosomal protein L21 (rpl21) is the conserved protein from cluster 6.

As a first step, we investigated the cluster distribution of the highly conserved rpoB protein (RNA polymerase subunit). Several features of rpoB including its universal presence in prokaryotes, its housekeeping function and its linear-inheritance constitute rpoB an ideal marker [23]. The rpoB protein is found in all four bacterial species analyzed here, and with the exception of *Buchnera aphidicola *rpoB protein, it is classified into the largest genomic group of all species (clusters 1, 2 and 6, Figure 3). For each of the clusters containing the rpoB protein, we have verified that its rank-BLAST vector corresponds to his taxonomic affiliation. For each of the three clusters, we constructed its rank-BLAST profile by calculating the mean position in the cluster of each of the 243 database species. In Figure 4, the rank-BLAST vectors are represented as barcodes, colored according to taxonomic relatedness. The rank-BLAST profiles of the clusters are compared to the profiles of conserved ribosomal proteins from each cluster (Figure 4). The comparison indicates that the clusters as a whole exhibit almost the same linear pattern of evolution as the conserved proteins. The barcodes confirm that the profiles of these clusters carry a phylogenetic signal, where species on the vector are ordered according to their phylogenetic proximity. Therefore these clusters are termed here 'main' clusters being both the largest genomic group in the corresponding species, and the carriers of a signal for a linear pattern of evolution.

This phylogenetic signal together with the classification of the conserved proteins into different genomic groups indicate that rank-BLAST profiles are indeed suitable for taxonomic stratification of genes, unlike other sequence-similarity based classification approaches such as phylogenetic profiles that are optimally designed to capture a functional signal [12,24,25]. A functional analysis of the main clusters further supports the strength of the approach in delineating a taxonomic, rather than functional, signal. The classification of proteins from the main clusters into functional categories is shown in Additional File 2, where many of the proteins are classified into highly-conserved categories such as transcription and ribosomal structure. The remarkable similarity observed between the patterns of functional distribution of proteins from *Mycoplasma genitalium *(cluster 2) and proteins from *Ureaplasma parvum *(cluster 6) - both members of the Mycoplasmataceae family - indicate that the rank-BLAST approach succeeds in correctly grouping proteins according to taxonomic origin and not according to functional similarity, even for orthologous proteins from closely related related species.

### Secondary genomic groups carry a signal for non-vertical evolution

For each species, we obtained more than a single genomic group (i.e. clusters dominated by proteins of the species). Whereas the inheritance pattern of the proteins in the main clusters corresponds to the species phylogeny, in secondary clusters we expect to detect different patterns, possibly patterns that correspond to events of non-vertical evolution of genes. For the purpose of identifying such patterns, we searched for clusters where we can define a ranking order which is common to the protein members of the cluster and is different from the ranking-order in the main cluster.

To identify secondary clusters with a coherent rank-BLAST profile, the mean ranking order of a species in a cluster was plotted against its number of appearances in the cluster (i.e., the number of protein members which recognize a homologue in the species and include it in their rank-BLAST profile). In Additional File 3, the two-dimensional representation of the cluster's rank-BLAST profile is shown for the four genomic-groups from *Streptococcus pyogenes*. Unlike the correlation observed between mean and number of appearances in the main cluster, inconsistent patterns are observed for the secondary clusters. In these clusters, many of the individual proteins differ in their rank-BLAST profiles from the rank-BLAST profile of the main cluster, though in most cases the differences are protein-specific rather than characteristic of a cluster. In order to estimate the coherence of the profile of each cluster, we compared the mean intra- and inter-cluster correlation in order to assess our ability to define a cluster-specific profile (Table 3). As described earlier for the main cluster, for each cluster the cluster-profile was initially constructed by calculating the mean position of each of the 243 database species in the cluster. Since many of the highly-ranked species in the secondary clusters appear only in a limited number of species [see Additional File 3] a reduced profile was also constructed which includes only widely-agreeable data points (see Table 3).

**Table 3:** Intra- and inter-cluster correlation in the four clusters dominated by *S. pyogenes *proteins.

	**Mean correlation between all protein-pair combinations†**				**Mean correlation with the cluster-profile††**				**Mean correlation with the reduced cluster-profile††**			
	**cl 1**	**cl 4**	**cl 14**	**cl 19**	**Cl 1**	**cl 4**	**cl 14**	**cl 19**	**cl 1**	**cl 4**	**cl 14**	**cl 19**
Cluster 1	**0.54**	0.30‡	0.40‡	0.14‡	**0.56**	0.57	0.41‡	034‡	**0.56**	0.52	0.40‡	034‡
Cluster 4	0.30‡	**0.51**	0.17‡	0.56	0.43‡	**0.53**	0.27‡	0.16‡	0.00	**0.04**	0.00	0.00
Cluster 14	0.40	0.17‡	**0.39**	0.18‡	0.44	0.17‡	**0.48**	0.27‡	0.47	0.18‡	**0.49**	0.28‡
Cluster 19	0.14‡	0.56	0.18‡	**0.32**	0.22‡	0.43	0.29‡	**0.47**	0.25‡	0.43	0.31‡	**0.47**

Intra-cluster combinations are shown in bold.

† Each cell shows the mean correlation for all possible protein-pair combination between the protein-members of the row-cluster and the protein-members of the column-cluster. Diagonal values show the intra-cluster correlation.

†† Each cell shows the mean correlation between cluster-profile of the row-cluster and all members of the column-cluster.

ℑ Reduced cluster profile is composed solely of data points defined as highly-agreeable. Agreeability was calculated for each data point (species) in a cluster as the inverse coefficient of variation weighted by the fraction of appearances of a species in a cluster:

*Agr *= (mean*app)/(sd*mmb)

Where *mean *is the mean position of a species in a cluster; app is the number of appearances of a species in a cluster; *sd *is the standard deviation calculated for the species; and *mmb *is number of proteins in a cluster.

Data points for which *Agr *> 0.5 are considered highly agreeable.

‡ The inter-cluster correlation is significantly lower than the intra-cluster correlation observed for the row-cluster. Significance is defined as *P *value < 0.05 in the Wilcoxon rank sum test (equivalent to the Mann-Whitney test).

**Figure 5:**
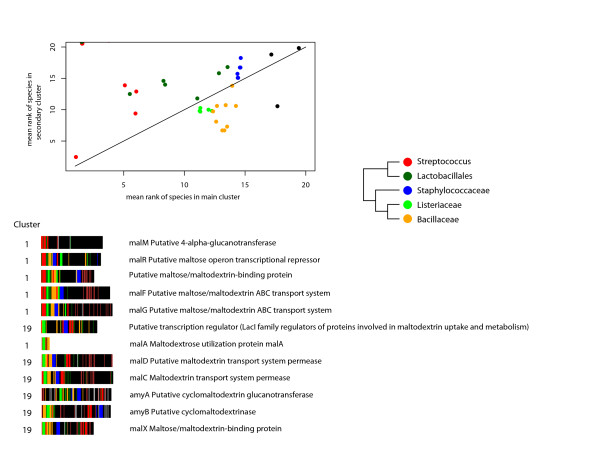
**Differences in the rank-BLAST profile between the main cluster (cluster 1) and secondary cluster (cluster 19) from *Streptococcus pyogenes***. (A) The ranking-order of the species in the main cluster versus the top species in the secondary cluster. The phylogenetic tree shows the taxonomic relationship between Bacilli groups. (B) Barcode representations of *Streptococcus pyogenes *proteins from cluster 1 and cluster 19 which are involved in transfer, metabolism and regulation of Maltose/maltodextrin. The order of the proteins corresponds with the genomic organization of their encoding genes. The color code is the same as in A.

From these results, it is observed that for secondary cluster 19 one can clearly define a cluster-profile which is common to the members of the cluster and is different from the profile of the main cluster (cluster 1). More specifically, the profiles of proteins from cluster 19 are significantly better correlated with the cluster-profile of cluster 19 than with the cluster-profile of cluster 1, and vice versa. We therefore focused on cluster 19 as a case study for comparing the profile of a secondary cluster to the profile of the main cluster while aiming to delineate the biological significance of this difference. The comparison of the cluster-profiles of cluster 1 and cluster 19 delineates a shift in the ranking order of Bacillales species and Lactobacillales species (Figure 5A). Whereas a higher rank of Lactobacillales species is expected from the taxonomic affiliation of *Streptococcus pyogenes*, and as observed for proteins from cluster 1, proteins from cluster 19 exhibit a higher similarity to Bacillales species. The ranking order of more distant species is consistent with the pattern observed in the main cluster (cluster 1), which is expected according to the phylogenetic classification of the species (not shown).

Individual examples for proteins from cluster 19 include a group of neighbouring protein-encoding genes that are involved in the uptake and utilization of maltosaccharide, a crucial process for the successful infection of *S. pyogenes *[26,27]. Members of this group exhibit not only increased sequence similarity within Bacillales species, but also similar genomic organization. The proteins are listed in Figure 5B according to the genomic order of their encoding genes. Whereas in other *Streptococcus *species malX, malC and malD are adjacent proteins and members of the *malXCD *operon [28], in *S. pyogenes *amyA and amyB are located between malC and malX. Interestingly, similar organization of orthologous proteins is also observed in Bacillales species such as *Geobacillus kaustophilus *(rather than in Lactobacillales). Another member of cluster 19 (Spy1297), a putative transcription regulator of proteins involved in maltose/maltodextrin uptake [29], is located upstream of these genes. Overall, the six proteins from cluster 19 together with additional proteins from cluster 1 form a genomic sequence of 12 proteins involved in maltose/maltodextrin uptake, metabolism and regulation. The rank-BLAST profile of these proteins is shown in Figure 5B. The proteins from cluster 1 demonstrate the expected phylogenetic order where they exhibit higher similarity to other Lactobacillales proteins, and present the same genomic organization reported in *Streptococcus *species [28]. The proteins from cluster 19 exhibit higher similarity to Bacillales, as well as a similar organization on the genome and correlated expression [27]. This similarity to Bacillales species (higher than the similarity to other Streptococci) suggests a common event of horizontal gene transfer (HGT) of the maltose related genes in cluster 19 [30], and a recent modification in the maltodextrin metabolism in *S. pyogenes*. Considering the role of maltodextrin acquisition in infecting host tissues [26,27], this modification is likely to have an adaptive advantage. The classification of all proteins from cluster 19 as well as other secondary clusters into functional categories is shown at Additional File 4.

### The rank-BLAST profile carries a genomic signature beyond the best-hit

**Figure 6:**
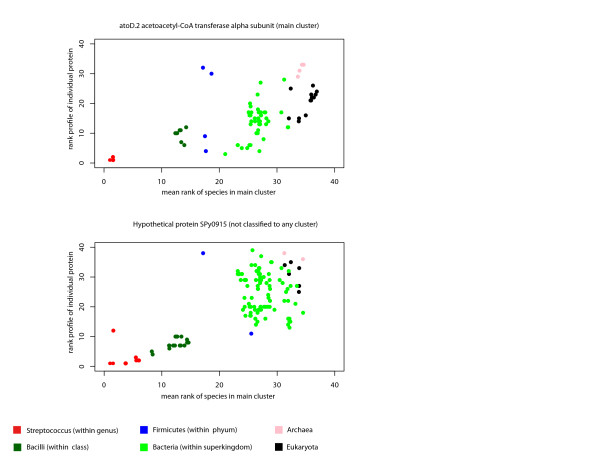
**The individual rank-BLAST profiles of two proteins from *Streptococcus pyogenes *classified into the main cluster (A) and not classified into any genomic group (B) versus the mean rank of species in the main cluster**.

Cluster 19 provides a compelling example of the ability of the rank-BLAST approach to group together genes with a common, non-vertical inheritance pattern. Since alternative approaches for delineating events of non-vertical evolution include the analysis of a typical sequence signature and the taxonomic identity of the BLAST best-hit [30], we have analyzed the distribution of best-hit partner and signature-based HGT events in the different genomic-groups, predicted for *S. pyogenes *(Table 4). The distribution of predicted HGT events in the main genomic group, secondary genomic groups and a group of proteins which were not classified to any genomic group (Table 4) indicates that no clear overlap can be specified between the signature-based predictions of non-vertical inheritance and the groups formed using the rank-BLAST procedure. This observation is not surprising, considering the low overlap between different methods for HGT predictions [31]. Similarly, the distribution of the BLAST best-hit partner in the different genomic groups reveals that despite different tendencies of the groups, the identity of the best-hit partner cannot justify the classification of proteins into clusters by itself. The best-hit distribution of *S. pyogenes *proteins is detailed at Figure 4. Overall, 27% of the proteins classified into a genomic group have a best-hit partner outside the Streptococcus genus. As shown at Table 4, for some of the proteins in the main cluster, we detected a best-hit partner in a species that is not a member of the same genus, the same class, the same phylum, or even the same super-kingdom. Vice versa, for many of the proteins that were not classified to any genomic group, we detect a *Streptococcus *best-hit partner. Examples for a protein in the main cluster with a best-hit partner that belongs to a different phylum (non-firmicutes species), and for a protein with a *Streptococcus *best-hit partner that was not classified into any genomic group are shown in Figure 6. Although the protein from the main cluster (Figure 6, top) identifies homologues in a limited number of non-firmicutes and non-Bacilli species, the ranking order of more distant species captures the same pattern as observed for more conserved proteins in the main cluster. In contrast, although the unclassified protein (Figure 6, bottom) captures the conservative ranking order of the highly-related species (genus, family members), the similarity of the profile is lost in more distant species. These examples, together with the distribution pattern in Table 4 indicate that the rank-BLAST profile captures a signal which is more informative than the best-hit partner. The ability of the approach to represent a more distant taxonomic signal is of special importance for the classification of sequences from metagenomic samples, which are in many cases only distantly related to sequences in the curated databases.

**Table 4:** Distribution of BLAST best-hit and sequence-signature based methods for prediction of HGT events.

			**Phylogenetic distribution of BLAST best-hit**					
			
		**No. of proteins†**	**Streptococcus Species^1^**	**Bacilli species^2^**	**Firmicutes Species^3^**	**Bacteria Species^4^**	**Archaea Species**	**Eukaryota Species**
***Main genomic group***	Cluster 1	1313 (225,95)	1101 (173,70)	146 (70,13)	22 (7,6)	28 (7,5)	1 (0,1)	3 (1,0)
***Secondary genomic groups***	Total	154 (35,18)	31 (9,2)	22 (6,3)	9 (6,4)	10 (4,2)	2 (0,1)	1 (0,0)
	Cluster 4	125 (23,14)	19 (6,0)	14 (1,2)	5 (4,3)	7 (2,2)	2 (0,1)	1 (0,0)
	Cluster 14	18 (8,2)	9 (2,1)	4 (3,0)	2 (2,1)	2 (1,0)	0	0
	Cluster 19	11 (4,2)	3 (1,1)	4 (2,1)	2 (0,0)	1 (1,0)	0	0
***Not in genomic groups***		239 (67,23)	58 (16,3)	86 (21,13)	9 (4,1)	21 (4,0)	3 (1,2)	3 (1,0)

In brackets: predictions for HGT events based on sequence-signature, retrieved from two public data sources: right - [43]; left - [44].

^1 ^other than *S. pyogenes; *^2^other than Streptococcus; ^3 ^other than Bacilli; ^4^other than Bacteria;

† Since some proteins recognize homologues only in strains of *S. pyogenes*, the number of proteins in cluster might be higher than the sum of the four columns on the right.

## Discussion

Here, we describe a novel approach for the assignment of protein sequences into genomic groups, using a simulation on a synthetic dataset composed of an assortment of proteins lacking taxonomic classification, originally retrieved from five species belonging to different taxonomic classes, orders and domains. The necessity of developing strategies towards taxonomic classification is relatively recent and arose as a result of the growing number of metagenomic projects. The annotation of sequences from metagenomic samples can be viewed as a two-dimensional task, i.e., whereas the annotation of species-specific genomes primarily concerns functional assignments, the annotation of metagenomic samples needs to provide taxonomic assignments as well.

Early annotation approaches before the sequencing of complete genomes used the best-hit of a sequence similarity search to infer function. The accumulation of a growing number of fully sequenced genomes has led to a shift of focus from gene-centric approaches to genomic-centric approaches. One such prominent genomic-centric approach is the phylogenetic profiles method, which analyses the distribution pattern of genes across genomes in order to retrieve a functional signal. Analogously, whereas basic approaches for taxonomic prediction rely on the identity of the best-hit [32], the rank-BLAST approach described here aims to maximize the information from a BLAST search in order to delineate a strong and consistent, species-specific phylogenetic signal. Our analysis indicates that the use of the rank-BLAST approach indeed allows an accurate taxonomic classification in cases where BLAST search by itself is not equally sensitive: whereas the rank-BLAST procedure allows the taxonomic classification at the species level of 64% of the proteins analyzed in this study, only 56% of the proteins had recognized a BLAST best-hit partner within the same genus. In *S. pyogenes *for example, 27% of the proteins classified into a genomic group have a best-hit partner outside the Streptococcus genus and even outside the class, the phylum, and super-kingdom (Table 4), indicating that the rank-BLAST procedure can outperform the basic BLAST search in assigning species into genomic groups.

Due to the complexity of the data analyzed, the retrieval of a taxonomic or functional signal from the results of a sequence similarity search is not trivial. Since first suggested in 1999 [12], the use of phylogenetic profiles had been extensively modified and optimized [24,25,33]. Similarly, the retrieval of phylogenetic signal is not a straightforward exercise and it requires the consideration of a large number of parameters, where several approaches can possibly be accounted for in each step of the procedure [34]. As part of the development of the rank-BLAST classification procedure, we have tested many alternatives in order to optimize the procedure, and examined their implications on the efficiency of clustering the data into genomic groups. Several approaches were tested for the following key steps: the construction of the vector describing the ranking-order of species; scoring the level of similarity between vectors; clustering the data; and the choice of query and database species. In order to emphasize the variety of options that need to be explored in each step, in the following paragraph we provide a brief description of some of the possibilities tested along these key steps. The construction of the vector best describing the ranking order of species was performed both according to bit-score [35] and E-value estimates for the BLAST search. Several possibilities for binning the data in different resolutions of the estimates were tested. Sequence similarity detection was based exclusively on BLAST. For the comparison of vectors we used several methods of correlation comparison, including Kendall's tau correlation, Spearman's correlation and Pearson correlation, and examined the intra-/inter-genomic stratification under the use of both the correlation estimates and *P *values. Other than correlation-based methods, we have also estimated the similarity of the ranking-order using decision-Trees and Bi-clustering approaches (not shown). For clustering of the data, we tested the results using k-means and a range of Machine Learning algorithms. The analysis was performed for five different species which represent varying taxonomic relationships between themselves, and with respect to the database species. It is likely that the differences observed in the clustering efficiency of the different genomes are related to the differences in phylogenetic resolution of the database. Although it is beyond the scope of the current work, studying the effect of the database (and reference genomes) is a future avenue of research.

As part of accuracy considerations, the sensitivity (allowing the classification of as many proteins as possible) and specificity (receiving as coherent genomic groups as possible; i.e., clearly relating to a single species) of the classification procedure were tested under different parameters. Since it is now widely accepted that a genome represents a dynamic collection of genes, whose composition may change across lineages, the clustering of all proteins of a species into a single genomic group was not a main consideration, assuming that different genes in the genome have followed different evolutionary paths. From the variety of approaches taken, the optimal procedure in terms of precision and recall yielded 20 genomic clusters, which almost uniquely represent a single species, and encompasses the large majority of the proteins in the analysis (Figure 3, as described in the text). The procedure enables the full separation between species within the same family (the two Mycoplasmataceae species *Mycoplasma genitalium *and *Ureaplasma parvum*). The different genomes were reconstructed with different efficiencies, where reconstruction of the original genomes is up to 92% (Table 2). As expected, for all species we have retrieved more than a single group, where the variety of groups detected for each genome enable us to carefully view the signal detected by the rank-BLAST procedure. For most of the test genomes, we have identified a main cluster whose phylogeny corresponds to species phylogeny, therefore providing a reassurance that the rank-BLAST procedure enables us to capture an intra-genomic signal retrieved from a group of genes with a common, conserved inheritance pattern. Genes classified to secondary groups do not necessarily follow this pattern and have possibly taken a different evolutionary path, which might be common to all members of the group (see above for an example).

## Conclusion

In conclusion, our results indicate that the rank-BLAST classification procedure is indeed suitable for capturing an intra-genomic signal, which to a large extent allows the reconstruction of meaningful, species-specific genomic groups. In some cases the procedure also allows the stratification of the genes into groups reflecting their individual histories. The example of the mutual Bacillales origin of the genes involved in the maltodextrin metabolism demonstrates that common origins can be associated with common functions. We therefore propose that this novel approach provides the basis for revealing the association between groups of genes, contributing an interesting, fresh perspective of the complex genotype-phenotype relationship. The rank-BLAST procedure can therefore not only delineate a phylogenetic-signal, but can also detect genes that exhibit specific patterns of evolutionary change that diverge from the whole-genome profile.

In the study of metagenomic data, the rank-BLAST procedure has a role in both obtaining taxonomic assignments and characterizing the conservation level of different functional groups. Firstly, the procedure can be used for the reconstruction of species-specific genomic groups. The ability of the procedure to capture a taxonomic signal which is beyond the BLAST best-hit is of special importance for the appropriate phylogenetic-localization of genes which belong to distantly related species with respect to the collection of species with fully sequenced-genomes. Secondly, as different environments exhibit a typical functional fingerprint [36], it will be interesting to examine whether 'signal-less' proteins form a functional group characteristic of a given environment. We are currently applying the rank-BLAST procedure for the study of metagenomic data.

## Methods

### Estimating divergence distance

The protein sequences and annotations of the five fully-sequences species analyzed (Table 1) were retrieved from the DOE Joint Genome Institute website IMG . The evaluated divergence distances between species were derived according to the scores of the CLUSTALW pairwise alignments [37] between the 16S rRNA from each species and the 16S rRNA from *M. genitalium*. CLUSTALW (version 1.83) was run from the EBI website  using the default parameters [37]. Each alignment score was normalized from the score of the alignment of the 16S rRNA of *M. genitalium *against itself. For example, the self alignment of the 16S rRNA from *M. genitalium *yielded a score of 100, and the alignment of the 16S rRNA from *M. genitalium *against the 16S rRNA from *U. parvum *yielded a score of 83. The divergence distance of *M. genitalium *from itself was considered to be 0 (100-100) and the divergence distance between *M. genitalium *and *U. parvum *was 17 (100-83). In case of multiple copies of 16S RNA, the closest sequence in a species is considered. Divergence distance scores were also compared to scores obtained when using the greengenes website [38]: Aligned 16S RNA sequences from the five species studied were retrieved from the website and then pairwise compared via the website using default parameters. Alignments are done using NAST, distance matrix is calculated using the DNAML option of DNADIST (PHYLIP package) [38]. The same divergence order is obtained by both methods (Table 1).

### Constructing a rank-BLAST profile

All proteins in the selected synthetic dataset were subject to a BLAST search (default BLAST parameters) against a collection of fully sequenced species. The collection of the complete genomes was retrieved from the COGENT database [39] representing the entire protein sequence complements from 243 species (including 197 Bacteria, 22 Archaea and 24 Eukaryota species). For each database protein (i.e., a protein coming from one of the five species in the analysis, as listed in Table 1) we used the scores of the BLAST search against the query proteins to construct a vector of the corresponding query species, each species in the vector is represented by the highest BLAST score of a homologue. Species within each vector were ranked according to the order of their corresponding scores. Species for which the E-value of their recorded score (best match) is of the same order (the same exponent) were assigned to an identical ranking score. This was done in order to provide a buffer which reduces the effect of subtle changes (for example small differences in the ranking order of closely related strains) on the overall analysis.

### Calculating the Kendall tau rank correlation coefficient

The Kendall tau rank correlation coefficient is used to measure the degree of correspondence between two rankings [40]. The correlation coefficient was calculated using the R statistical platform.

### Computing the hypergeometric probability

In order to calculate the hypergeometric probability, the correlation matrix was first transformed into a binary matrix, where values of tau correlation coefficient > 0.7 were converted to 1 (positive events) and lower values were converted to 0. This cut-off was selected as a trade-off between achieving maximum intra-inter genomic separation and keeping as many "positive" correlations as possible (Figure 2).

The probability of observing at least *x *positive events in common between two different columns in the binary matrix was computed using the hypergeometric distribution, assuming that every pattern of *n *positive events is equally probable. In that case, if, in the binary matrix obtained from correlation coefficients, the total number of events (positive and negative) in columns A and B is *N*, and *n *and *D *are the number of positive events in A and B respectively, the probability of observing at least *x *common positive events by chance alone is:



### Generating genomic clusters

The P values for the hypergeometric probability are stored in memory and then written into five binary files, each of which represents a different threshold, so that they need not be recomputed for subsequent analysis. Each file describes a network which consists of proteins (nodes) connected by P values above the set threshold (edges). The five thresholds for the P values are: 1 × 10^-25^; 1 × 10^-50^; 1 × 10^-75^; 1 × 10^-100^; 1 × 10^-150. ^We use the MCL (**M**arkov **Cl**ustering) algorithm to cluster this network according to connectivity and local structure [41]. The inflation value parameter of the MCL algorithm is used to control the granularity of these clusters. The data was tested under inflation values ranging from 1 to 3. The use of different parameters for the P value threshold and for the inflation values yielded stable clusters (not shown). Optimal results (reported here) were obtained using P value threshold of 1 × 10^-75 ^with inflation value 1.8.

To examine the effect of genome size on the efficiency of the classification procedure we have studied the correlation between the number of proteins encoding genes in the species used in the analysis (Table 1) and the efficiency of the classification procedure (Table 2) - no such correlation is observed (Spearman's rho correlation = 0, *P *value = 1).

### Comparing the sensitivity of the taxonomic classification using the rank-BLAST approach to the sensitivity using BLAST best-hit partner

The rank-BLAST classification approach allows the classification of 2590 proteins (out of 3891) into genomic groups. From these, 2505 proteins are classified into a corresponding genomic group; that is protein A is classified into a corresponding genomic group if most proteins in the cluster are coming from the same species as protein A. The distribution of proteins within the genomic groups is listed at Figure 3. Sensitivity is calculated as the fraction of correct classifications in the entire data set of 3891 proteins (64%). The overall specificity of the genomic groups is calculated as the fraction of correct classifications out of all proteins classified into any genomic group (2590 proteins, as listed in Figure 3).

The pre-processed BLAST results of the 3891 proteins versus the database of complete genomes (as described at the *Constructing a rank-BLAST profile *sub-section) were used for estimating the sensitivity of the taxonomic classifications according to BLAST best-hit partners. Recognition of a BLAST best-hit partner within the same genus (excluding hits versus proteins of the corresponding species) is considered as a correct taxonomic classification. Sensitivity is calculated as the fraction of correct classifications in the entire data set of 3891 proteins (56%).

All software used for the analysis will be provided upon request from the authors.

## Authors' contributions

SF conceived and performed the analysis, and drafted the manuscript. LG was involved in all stages of work and examined several clustering approaches. AG tested various clustering approaches. EB contributed for performing the hypergeometric distribution analysis. ST has provided critical comments for the analysis and assisted in the organization and writing of the manuscript. CAO advised on the analysis, contributed with discussions of draft versions and assisted in the organization and writing of the manuscript. All authors read and approved the final manuscript.

## Supplementary Material

Additional file 1 **Supplementary notes**. A description of the efficiency of clustering achieved with alternative approaches. Click here for file

Additional file 2 **Supplementary Figure 1**. Functional distribution of proteins in COG clusters. Click here for file

Additional file 3 **Supplementary Figure 2**. Mean position in the cluster of each of the 243 database species versus the number of appearances of the species in the cluster. Click here for file

Additional file 4 **Supplementary Figure 3**. Functional distribution of proteins from the main and secondary genomic groups of *Streptococcus pyogenes *in COG clusters. Click here for file
