# Supplementary material for: Stratification of co-evolving genomic groups using ranked phylogenetic profiles
Source: BMC Bioinformatics. 2009 Oct 27;10:355. doi: 10.1186/1471-2105-10-355 (PMC2775751; doi:10.1186/1471-2105-10-355)
Supplement: Additional file 1 — Supplementary notes. A description of the efficiency of clustering achieved with alternative approaches. [file 1471-2105-10-355-S1.pdf]

Stratification of co-evolving genomic groups using ranked phylogenetic profiles

Shiri Freilich[1,2†], Leon Goldovsky[1], Assaf Gottlieb[3], Eric Blanc[4,5], Sophia Tsoka[4], Christos A. Ouzounis[1,4†]

Testing alternative clustering approaches

We compared the results obtained by the approach taken in the main text to the results obtained from two different clustering algorithms: the *k*-means clustering algorithm, (operating on the hypergeometric probability matrix) and biclustering on a binary representation of the ranked-BLAST profile. All algorithms were performed using Matlab.

Description of methods

K-means algorithm

We first preprocessed the hypergeometric probability matrix by applying Singular Value Decomposition (SVD) and took the first five left singular vectors. Removing the first left singular vector does not alter the results significantly. SVD is the same as applying Principal Components Analysis on the covariance matrix of the hypergeometric probability matrix [1].
The *k*-means clustering algorithm was used on the preprocessed data with k= 5, 10, 15 and 20. For each *k*, the results are averaged over 50 independent runs.

Biclustering algorithm

Biclustering was performed on the binary ranked-BLAST matrix. The threshold was set to 10 such that each protein having a rank less than 10 was given a 1 and 0 otherwise. We used a bipartite spectral graph partitioning algorithm of [2]. Initially designed for documents and words, this biclustering algorithm handles sparse data well. This algorithm produces biclusters of the 3891 test proteins and 243 species. We augmented the algorithm to produce good biclusters' images. This was achieved by

applying single linkage hierarchical algorithm for each produced bicluster and sorting each bicluster according to the hierarchical clustering, thus handling less homogenous clusters better.

Results

In order to assess the purity of each cluster, we used a weighted Positive Predictive Value (PPV). Each calculated cluster may contain more than one of the original classes. We considered the most prevalent class (species) as the true class of this cluster and the number of the cluster members in the prevalent class as true positives. We calculated the fraction of the true positives over all cluster member (true positives and false positives), which is the PPV value. Normalizing the results per cluster with cluster size, the overall PPV of a clustering result is the sum of all true positives over all the dataset. Table S1 lists the average PPV over all independent runs for each *k*, compared with the results of the Markov Clustering (MCL) in the article. The PPV was calculated both for all the data and to the subset found by the MCL algorithm. We see that the MCL algorithm outperforms the *k*-means, where its clusters are almost pure, i.e. each contains almost exclusively a single class. The results are better for higher *k*.

We also calculated the weighted Jaccard score for each of the clustering parameters. Jaccard score is calculated as intersection between cluster members and class members over union of these members and ranges from 0- no match to 1- prefect match between clusters and classes. The weighted Jaccard score uses the same prevalent class for calculating the score and normalizes by class size. The weighted Jaccard score also takes into account the false negatives, which PPV ignores. The average results over the independent runs are shown in table S2. In table S2 we see that the results of *k* higher than five are inferior. We also observe that the results for *k*-means with *k*=5 and the Biclustering for k=6 and 7 are slightly better than the MCL algorithm, when calculating only on the subset of proteins used by the MCL algorithm. This comes from the fact that the MCL algorithm focused on the positive predictive value of the clusters and not on the sensitivity of the clusters.

An example of the biclustering results can be seen in Figure S3 for six clusters. Figure S3 displays that the four clusters match almost exclusively a single species class (sp, ba and ne). One cluster has equal numbers of *Mycoplasma genitalium* and

Ureaplasma parvum and one cluster is a mixture of mainly *Mycoplasma genitalium, Ureaplasma parvum* and *Streptococcus pyogenes*.

Both k-means and biclustering methods can distinguish between the tested species and species from the same phylum, super-kingdom and distinct super-kingdom, but are unable to clearly distinguish between members of the same family. A clear stratification of proteins belonging to species which are members of the same family was only obtained when using the MCL approach (as described in the main text).

Table S1. Average PPV for different clustering parameters. Best PPV is in bold.

**clustering method**	**all data (3891 proteins)**	**MCL clusters data (2591 proteins)**
MCL (20 clusters)	-	**0.97**
k-means (*k*=5)	0.76	0.84
k-means (*k*=10)	0.78	0.85
k-means (*k*=15)	0.81	0.86
k-means (*k*=20)	0.83	0.88
Biclustering (k=6)	0.78	0.80
Biclustering (k=7)	0.80	0.83
Biclustering (k=10)	0.78	0.77
Biclustering (k=15)	0.77	0.78
Biclustering (k=20)	0.76	0.75

Table S2. Average weighted Jaccard score for different clustering parameters. Best Jaccard is in bold.

**clustering method**	**all data (3891 proteins)**	**MCL clusters data (2591 proteins)**
MCL (20 clusters)	-	0.71
k-means (*k*=5)	0.65	**0.73**
k-means (*k*=10)	0.44	0.48
k-means (*k*=15)	0.33	0.38
k-means (*k*=20)	0.26	0.30

Biclustering (k=6)	0.67	**0.73**
Biclustering (k=7)	0.67	**0.73**
Biclustering (k=10)	0.53	0.56
Biclustering (k=15)	0.44	0.47
Biclustering (k=20)	0.35	0.36

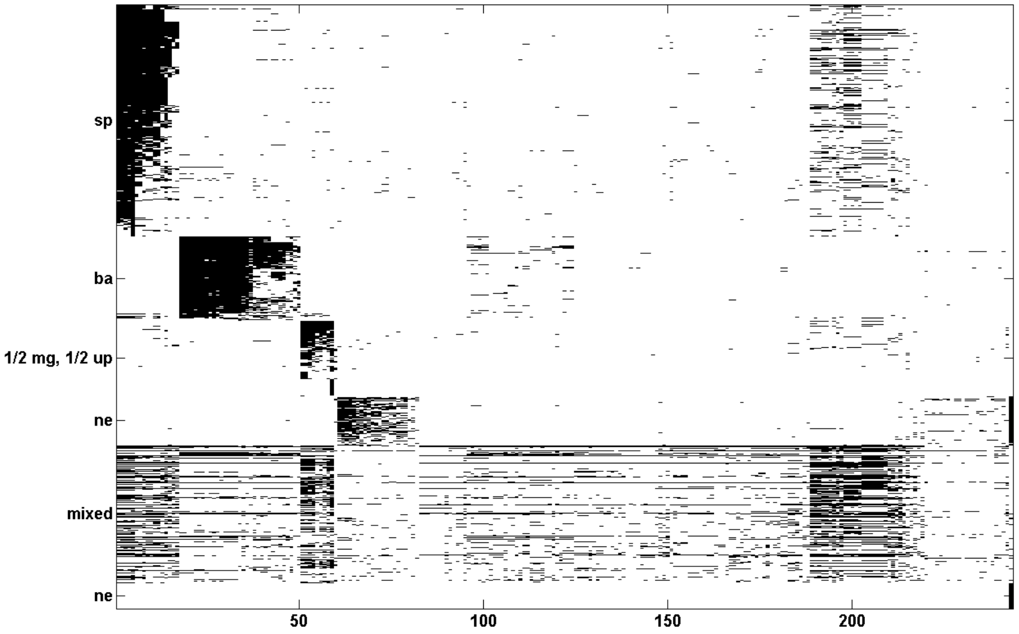

Figure S3 Biclustering proteins vs. database species. X-axis denote database species and y-axis denotes proteins, showing primary class name (mg= *Mycoplasma genitalium*, up= *Ureaplasma parvum*, sp= *Streptococcus pyogenes*, ba= *Buchnera aphidicola* and ne= *Nanoarchaeum equitans)*

1. Wall, M.E., P.A. Dyck, and T.S. Brettin, *SVDMAN--singular value decomposition analysis of microarray data.* Bioinformatics, 2001. **17**(6): p. 566-8.
2. Dhillon, I.S., *Co-clustering documents and words using bipartite spectral graph partitioning.* In Proceedings of the ACM SIGKDD Conference, 2001: p. 269-274

Supplementary Figure legends (files enclosed)

Supplementary Figure 1: Functional distribution of proteins in COG categories. Categories R, S, and X refer to poorly characterized sequences (general function

prediction, unknown function, and sequences not assigned to any COG category, respectively).

Supplementary Figure 2: Mean position in the cluster of each of the 243 database species versus the number of appearances of the species in the cluster (number of proteins in the cluster which recognize a homologue in the species). All clusters are dominated by proteins from *Streptococcus pyogenes*. The color code represents the phylogenetic proximity between the species in the vector (database species) and *Streptococcus pyogenes*. Red: Streptococcus species (within the same genus as *Streptococcus pyogenes*); Dark green: Bacilli species (within the same class); Blue: Firmicutes species (within the same phylum); Green: Bacteria species (within the same kingdom); Black: Archaea/Eukaryota species (different super-kingdom).

Supplementary Figure 3: Functional distribution of proteins from the main and secondary genomic groups of *Streptococcus pyogenes* in COG clusters. More than 80% of the proteins in cluster 4 are not classified into any functional category and hence the distribution of proteins from this cluster is not shown. Categories R, S, and X refer to poorly characterized sequences (general function prediction, unknown function, and sequences not assigned to any COG category, respectively).
