# Supplementary figures and images for: Stratification of co-evolving genomic groups using ranked phylogenetic profiles
Source: BMC Bioinformatics. 2009 Oct 27;10:355. doi: 10.1186/1471-2105-10-355 (PMC2775751; doi:10.1186/1471-2105-10-355)

Sup figure 1

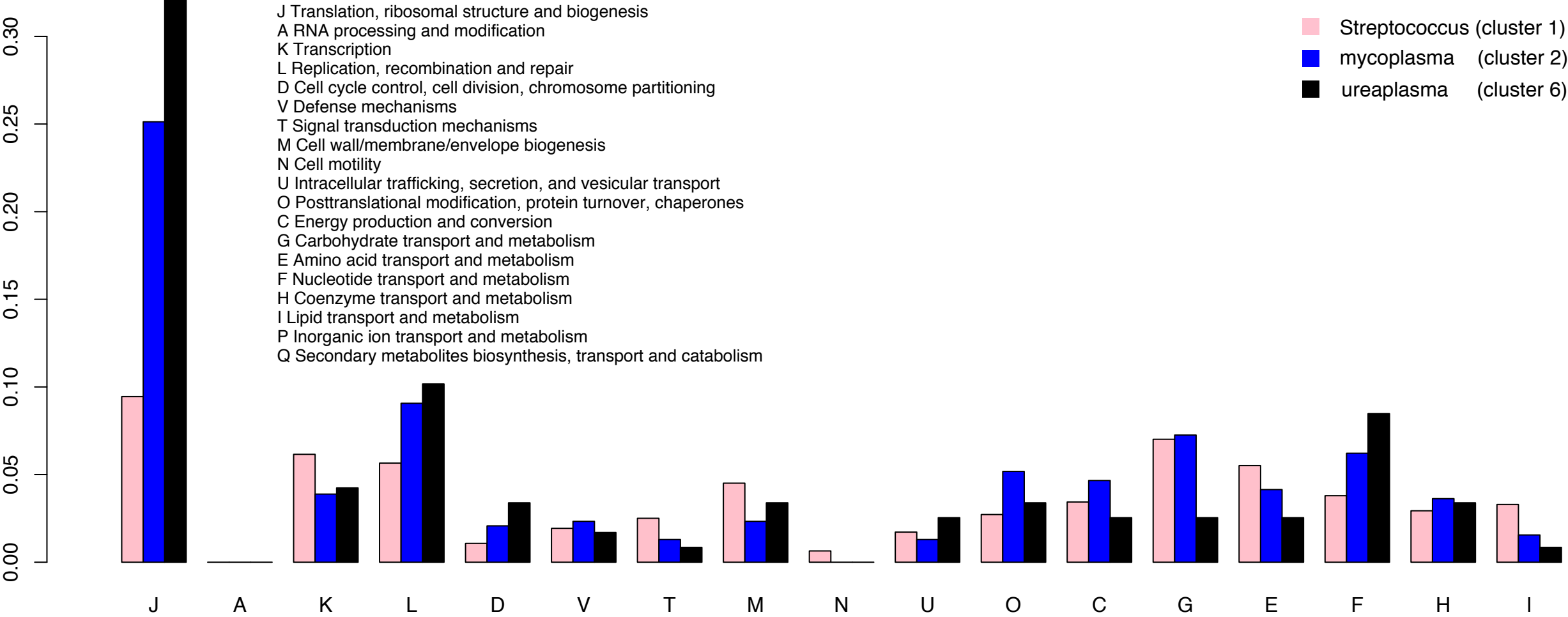

Supplement: Additional file 2 — Supplementary Figure 1. Functional distribution of proteins in COG clusters. [file 1471-2105-10-355-S2.pdf]

Sup. Figure 2

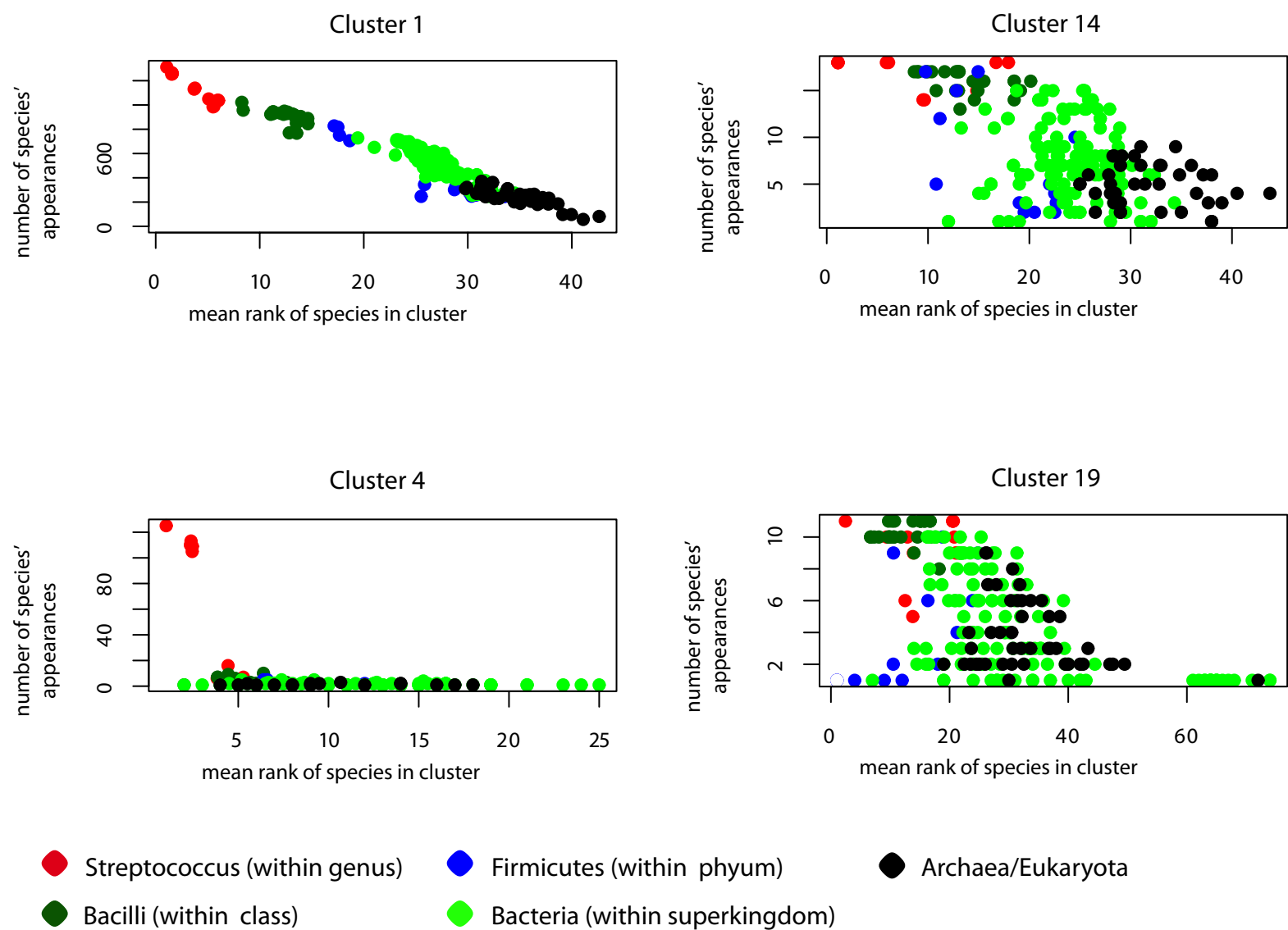

Supplement: Additional file 3 — Supplementary Figure 2. Mean position in the cluster of each of the 243 database species versus the number of appearances of the species in the cluster. [file 1471-2105-10-355-S3.pdf]

Sup Figure 3

sp clusters

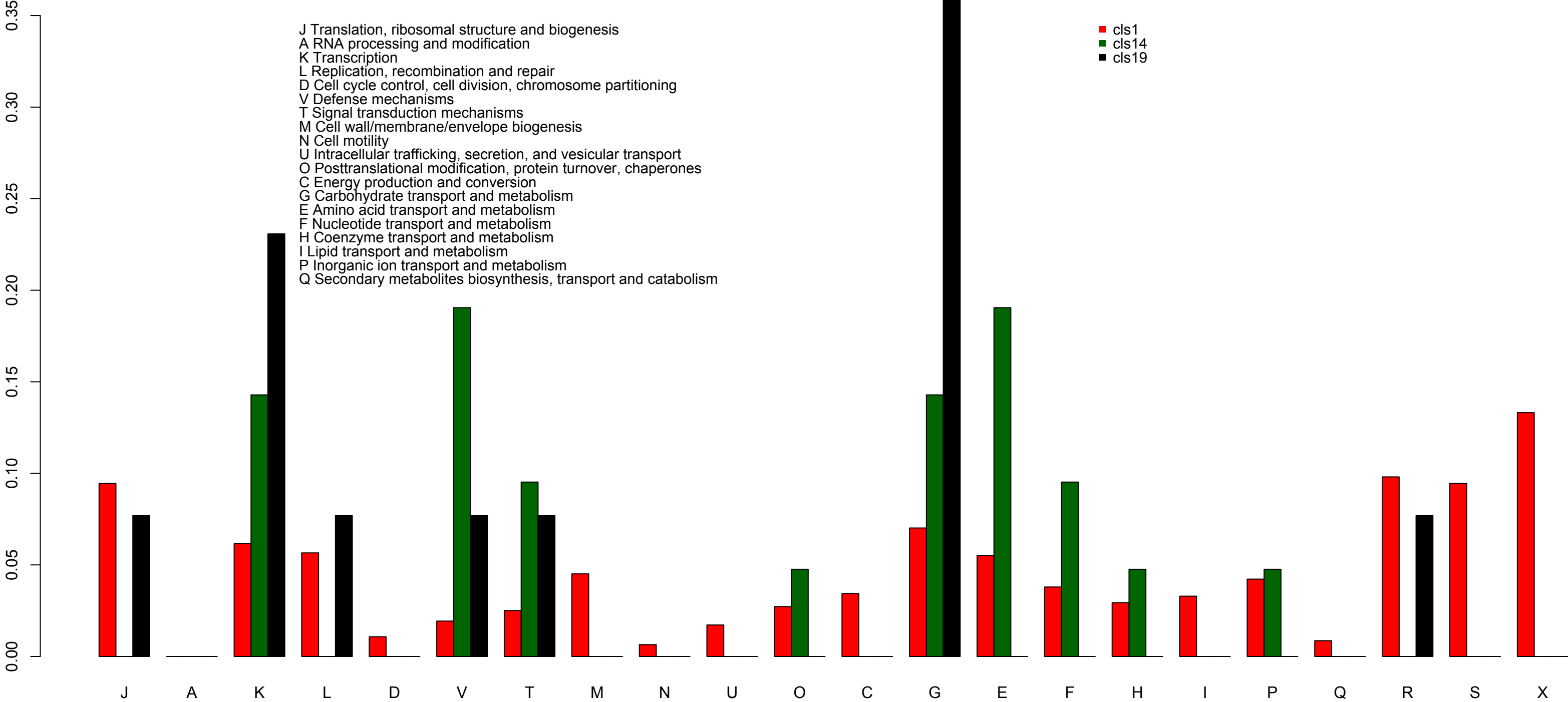

Supplement: Additional file 4 — Supplementary Figure 3. Functional distribution of proteins from the main and secondary genomic groups of Streptococcus pyogenes in COG clusters. [file 1471-2105-10-355-S4.pdf]
